# The Role of Multiple Re-Entry Tears in Type B Aortic Dissection Progression: A Longitudinal Study Using a Controlled Swine Model

**DOI:** 10.1177/15266028221111295

**Published:** 2022-07-19

**Authors:** Chlöe Armour, Baolei Guo, Simone Saitta, Daqiao Guo, Yifan Liu, Weiguo Fu, Zhihui Dong, Xiao Yun Xu

**Affiliations:** 1 Department of Chemical Engineering, Imperial College London, London, UK; 2 Department of Vascular Surgery, Zhongshan Hospital, Institute of Vascular Surgery, Fudan University, Shanghai, China; 3 Department of Vascular Surgery, Qingpu Branch of Zhongshan Hospital Affiliated to Fudan University, Shanghai, China; 4 Department of Electronics, Information and Bioengineering, Politecnico di Milano, Milan, Italy

**Keywords:** aortic dissection, re-entry tear, false lumen expansion, computational hemodynamics, 4D-flow MRI

## Abstract

**Purpose:**

False lumen (FL) expansion often occurs in type B aortic dissection (TBAD) and has been associated with the presence of re-entry tears. This longitudinal study aims to elucidate the role of re-entry tears in the progression of TBAD using a controlled swine model, by assessing aortic hemodynamics through combined imaging and computational modeling.

**Materials and Methods:**

A TBAD swine model with a primary entry tear at 7 cm distal to the left subclavian artery was created in a previous study. In the current study, reintervention was carried out in this swine model to induce 2 additional re-entry tears of approximately 5 mm in diameter. Computed tomography (CT) and 4-dimensional (4D) flow magnetic resonance imaging (MRI) scans were taken at multiple follow-ups before and after reintervention. Changes in aortic volume were measured on CT scans, and hemodynamic parameters were evaluated based on dynamic data acquired with 4D-flow MRI and computational fluid dynamics simulations incorporating all available in vivo data.

**Results:**

Morphological analysis showed FL growth of 20% following the initial TBAD—growth stabilized after the creation of additional tears and eventually FL volume reduced by 6%. Increasing the number of re-entry tears from 1 to 2 caused flow redistribution, with the percentage of true lumen (TL) flow increasing from 56% to 78%; altered local velocities; reduced wall shear stress surrounding the tears; and led to a reduction in FL pressure and pressure difference between the 2 lumina.

**Conclusion:**

This study combined extensive in vivo imaging data with sophisticated computational methods to show that additional re-entry tears can alter dissection hemodynamics through redistribution of flow between the TL and FL. This helps to reduce FL pressure, which could potentially stabilize aortic growth and lead to reversal of FL expansion. This work provides a starting point for further study into the use of fenestration in controlling undesirable FL expansion.

**Clinical Impact:**

Aortic growth and false lumen (FL) patency are associated with the presence of re-entry tears in type B aortic dissection (TBAD) patients. Guidelines on how to treat re-entry tears are lacking, especially with regards to the control and prevention of FL expansion. Through a combined imagining and computational hemodynamics study of a controlled swine model, we found that increasing the number of re-entry tears reduced FL pressure and cross lumen pressure difference, potentially stabilising aortic growth and leading to FL reduction. Our findings provide a starting point for further study into the use of fenestration in controlling undesirable FL expansion.

## Introduction

Type B aortic dissection (TBAD) is a complex disease of the aorta, in which a tear forms in the inner layer of the aortic wall, through which blood flows creating a secondary channel known as the false lumen (FL). Patients are typically treated either medically or with thoracic endovascular aortic repair (TEVAR), the latter involves inserting a stent-graft into the true lumen (TL) to seal the primary entry tear. Surgical treatment such as the frozen elephant trunk method may also be considered.^1^ It is common for TBAD patients to present with multiple re-entry tears.

A large number of anatomical studies have been conducted with an aim to identify morphological parameters that can predict TBAD progression and complications; however, an independent predictor of such complications has yet to be identified. A parameter of interest highlighted in several of these anatomical studies is the number of re-entry tears, with the number of tears linked to aortic growth^2–4^ and the lack of FL thrombosis^5^ in medically treated patients. Although the evidence is clear that the presence of re-entry tears reduces FL thrombosis,^5,6^ there are mixed conclusions with regard to aortic growth. Kotelis et al^2^ reported that an increase in the number of tears increased the risk of aortic growth, whereas Tolenaar et al^3,4^ reported a reduction in aortic growth with an increasing number of tears.

Both idealized and patient-specific computational and experimental studies have found that increasing tear numbers can reduce flow reversal throughout the aorta,^7^ reduce FL pressure,^8,9^ increase TL pressure,^10^ and equalize TL/FL cross-lumen pressure differences (CLPDs).^11,12^ Although providing insightful information on hemodynamic changes due to re-entry tears, these studies were limited by their lack of patient-specific inlet and outlet boundary conditions.

The effect of tear number and configuration was also examined in ex vivo experimental studies^13,14^ but these were limited by short experimental time scales and the often-non-physiological blood substitute used. In vivo animal studies^15,16^ have aimed to overcome such limitations; however, long-term studies are lacking.

Considering the current literature and the lack of well-controlled in vivo longitudinal animal models, the aim of this study is to gain a comprehensive understanding of the role of re-entry tears in aortic hemodynamics in TBAD through a longitudinal study of a controlled swine model, by using and combining extensive medical imaging with image-based computational modeling. The combination of 4-dimensional (4D) magnetic resonance imaging (MRI) and computational analysis of an in vivo TBAD model with longitudinal follow-ups, not previously employed in studies of re-entry tears, will aid in understanding the conflicting conclusions of previous anatomical studies^2–4^ regarding the role of re-entry tears in disease progression.

## Materials and Methods

**Figure 1.**
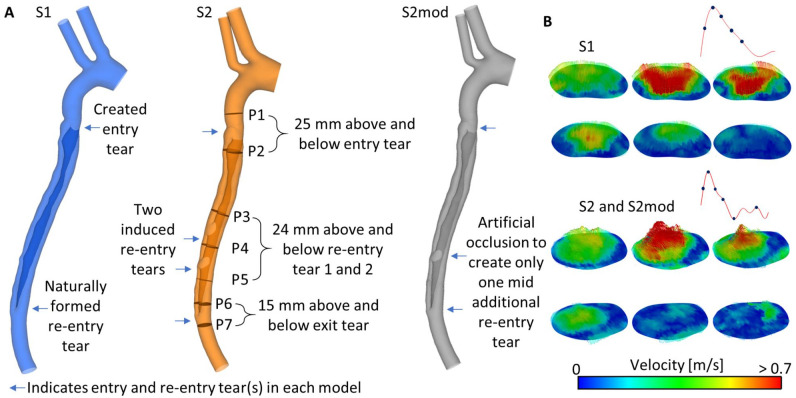
(A) Geometry of S1, S2, and S2mod. Analysis planes (P1-7) are indicated on S1. (B) 3D inlet velocity profile for S1, S2, and S2mod, extracted from 4D-flow magnetic resonance imaging.

One of the TBAD swine models reported previously^15^ was selected for further analysis and used in the present study. Only a single swine (male, 68.5 kg, 4 months old) was included in this study and no other animals underwent the protocol described in the following section. The study was approved by the Institutional Animal Care and Use Committee of Fudan University, China (approval reference number Y2014-138). All procedures conformed to ARRIVE guidelines and the guidelines from Directive 2010/63/EU of the European Parliament on the protection of animals used for scientific purposes. In the original study,^15^ following the creation of the TBAD 1 re-entry tear naturally formed 25 cm below the primary entry tear. Twenty months after the initial TBAD, reintervention was carried out and 2 additional re-entry tears were created in the middle section of the dissected aorta, resulting in a total of 4 tears. The distance from the left subclavian artery to the primary entry tear, additional re-entry tear 1, additional re-entry tear 2, and naturally formed re-entry tear was 7, 21, 26, and 32 cm, respectively. The location of each of these tears is indicated in Figure 1A.

### Creation of Additional Re-Entry Tears in TBAD Swine Model

The swine was placed in supine position under general anesthesia following the same procedure previously reported.^15^ The swine was sedated with an intramuscular injection of ketamine hydrochloride (15 mm/kg) and atropine sulfate (0.04 mg/kg). General anesthesia was induced with isoflurane (5%) administered with a face mask and a target-controlled infusion (TCI) of remifentanil at 4 ng/mL. To facilitate endotracheal intubation, rocuronium was given at 0.5 mg/ kg. After intubation, anesthesia was maintained with isoflurane (1.5e3%), oxygen (0.8e1.5 L/min), and mechanical ventilation. The TCI of remifentanil was reduced to match the level of surgical stimulation. Invasive blood pressure monitoring was established in the left femoral artery.

A 0.035-in guidewire (Terumo, Tokyo, Japan) and a 4F pigtail catheter were advanced into the thoracic and abdominal aorta to allow a full angiographic evaluation of the aorta through an introducer sheath (6F) in the right femoral artery. Surgical exposure of the right common carotid artery enabled the insertion of a steerable 8F, 55 cm Fustar sheath (Lifetech Scientific Inc, Shenzhen, China). A needle puncture system^17^ in conjunction with the Fustar sheath was then delivered to the FL. By holding the needle perpendicular to the dissection flap with the assistance of the steerable sheath, a hole was created in the dissection flap. The aperture was sequentially dilated with balloon catheters to the desired size (5 mm) of re-entry tear. The final angiogram and intravascular ultrasound (IVUS) were performed to evaluate the newly created tear to ensure it was of the desired size. This procedure was carried out twice to create the 2 additional tears. The puncture point of common carotid artery was sutured with 6-0 Prolene. The sheath in the femoral artery was removed and hemostasis was achieved by manual compression. The animal was extubated and returned to their cage.

### Imaging Protocol

Computed tomography (CT) and 4D-flow MRI scans were taken at several time points as described in Table 1. The 4D-flow MRI was performed using a 3T clinical MRI scanner (Magnetom Verio, Siemens Medical Solutions, Erlangen, Germany) with the following parameter setting: flip angle, 7°; velocity encoding, 150 cm/s; spatial resolution, (1.875-2.5) × (1.875-2.5) × 2.5 mm^3^; temporal resolution, 39.2 ms; and 14-25 frames/cardiac cycle. Retrospective electrocardiographic gating was used to synchronize the measurement with the cardiac motion. The CT scans were performed with the Aquilion (Toshiba, Canon Medical Systems, Zoetermeer, the Netherlands) scanner, with a spatial resolution of 0.8 × (0.892-0.961) × (0.892-0.961) mm^3^ and a kVp of 120.

**Table 1.** Information on the Evolution of TBAD Model.

Scan	State of model
	TBAD created
Scan 0 (S0)	1 month after the creation of TBAD
Scan 1 (S1)	9 months after the creation of TBAD
	Additional re-entry tears created
Scan 2 (S2)	1 month after reintervention
Scan 3 (S3)	5 months after reintervention
Scan 4 (S4)	9 months after reintervention

Abbreviation: TBAD, type B aortic dissection.

### Geometry Reconstruction

The CT scans were processed using Mimics (Materialize, Leuven, Belgium) to reconstruct geometric models of the aorta at different time points. Volumes of the TL and FL were calculated based on the segmented area of each slice and slice thickness. Two scans, S1 and S2, were chosen for computational fluid dynamics (CFD) analysis. Furthermore, an additional geometry, S2mod, was created in which the first re-entry tear in S2 was artificially occluded, thereby creating a model with only 1 additional tear. The final geometries of S1, S2, and S2mod are shown in Figure 1A. The models were meshed in ICEM (v15; Ansys Inc, Canonsburg, Pennsylvania). Mesh sensitivity tests were conducted to ensure mesh independence. The final meshes contained 3.9, 5.6, and 5.6 million elements for models S1, S2, and S2mod, respectively.

### Computational Details

Three-dimensional (3D) inlet velocity profiles (Figure 1B) were extracted from the 4D-flow MRI data acquired at S1 and S2 using an in-house MATLAB tool. To ensure the computational results were physiologically correct, 3-element Windkessel models were adopted as outlet boundary conditions.^18^ The Windkessel parameters were tuned using flow rates derived from the corresponding 4D-flow MRI data (S1 or S2) and Doppler-wire pressure measurements taken during S3. Flow rates were estimated by calculating the difference in flow immediately before and after the arch branches and splitting this flow based on the cross-sectional area of each branch.

All simulations were run in Ansys CFX (v15). The blood was assumed to have a constant density of 1022 kg.m^−3^ and was modeled as a non-Newtonian fluid dictated by equation 1, where η is the viscosity, $\dot{\gamma }$ is the shear rate, $K=0.08\;\mathrm{Pa}{\mathrm{.s}}^{n}$ and n=0.55 :^19^

(1) $$\eta =K\times {\dot{\gamma }}^{n-1}.$$

The wall was assumed to be rigid, flow was assumed to be laminar, a time-step of 0.001 s was used, and all simulations were run until a periodic solution was reached. An analysis of the computational results was performed in EnSight. Seven planes (P1-P7) placed perpendicular to the wall along the centerline were selected for detailed analysis and these are defined in Figure 1A (in model S2) along with distances relative to nearby tears. Flow distribution, reverse flow index^7^ (RFI, equation 2), velocity, time-averaged wall shear stress (TAWSS), and pressure were evaluated for all models. Flow distribution, velocity, and pressure are key parameters that describe the hemodynamic state of the dissection and are useful for quantitative comparisons between the TL and FL and between different models. The RFI measures the percentage of flow that is moving in the opposite direction (quantifying the amount of regurgitant flow). This is important to assess as regurgitant flow can indicate flow stagnation that favors platelet adhesion and thrombus formation. Evaluating TAWSS is also important as it indicates the level of frictional stress exerted on the endothelial lining of the vessel wall. Around the tears, high TAWSS levels may cause the tear to grow, whereas high TAWSS in the FL may reduce the likelihood for thrombus formation:

(2) $$\mathrm{RFI}=100\%\times \frac{\left|\int_{0}^{T}Q_{\mathrm{reverse}}dt\right|}{\left|\int_{0}^{T}Q_{\mathrm{reverse}}dt\right|+\left|\int_{0}^{T}Q_{\mathrm{forward}}dt\right|}.$$

## Results

### Morphological Changes

Table 2 shows the percentage change in TL and FL volumes between each scan, and the dimensions of the entry and distal re-entry tears at each point. Large expansions of the TL and FL were observed between S0 and S1, and S1 and S2. After the additional tears were created (S2 onward), FL expansion slowed (S2-S3) and eventually reversed (S3-S4). The entry tear did not significantly change in size, whereas the naturally formed distal re-entry tear slowly expanded between S0 and S2.

**Table 2.** Top—Changes in TL and FL Volume Between Follow-up Scans. Bottom—Maximum Axial Diameter of Entry and Distal Tears.

	TL volume change (%)		FL volume change (%)
S0 → S1	12.4		3.6
S1 → S2	14.1		19.7
S2 → S3	2.5		0.6
S3 → S4	–11.8		–6.2
S0 → S3	17.1		20.3
S0 → S4	3.1		13.0
	TL max diameter (mm)	FL max diameter (mm)	Aorta max diameter (mm)
S0	17.3	20.8	27.4
S1	19.4	21.7	31.4
S2	21.1	23.1	31.3
S3	19.5	22.3	30.3
	Entry tear max axial diameter (mm)		Distal tear max axial diameter (mm)
S0	19.3		8.8
S1	21.0		9.5
S2	21.8		12.1
S3	21.5		11.6

Abbreviations: FL, false lumen; TL, true lumen.

### Hemodynamic Changes

**Figure 2.**
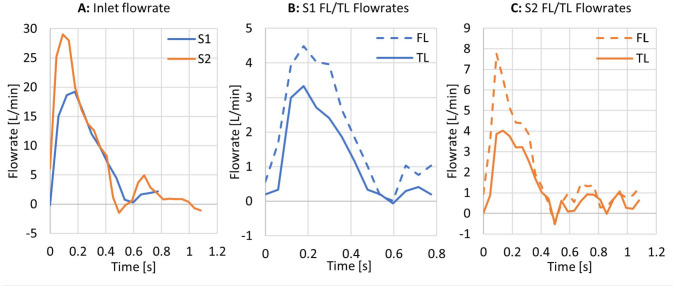
(A) Inlet flow rate extracted from 4-dimensional flow magnetic resonance imaging (4D-flow MRI). (B and C) TL and FL flow rates extracted from 4D-flow MRI. TL and FL flow rates were evaluated on plane 2, location of which is shown in Figure 1A. TL, true lumen; FL, false lumen.

Figure 2 shows the volumetric flow rates (measured on plane 2 for TL/FL values) derived from the 4D-flow MRI data acquired at S1 and S2. It can be seen that the cardiac cycle at S1 (0.78 seconds) was shorter than that at S2 (1.08 seconds), whereas S2 had a higher peak inlet flow rate, and S1 a shorter diastolic phase. On both occasions, FL flow was higher than flow in the TL. S2 saw a higher proportion of inlet flow reporting to the descending aorta (48.6%) compared with S1 (34.9%).

**Figure 3.**
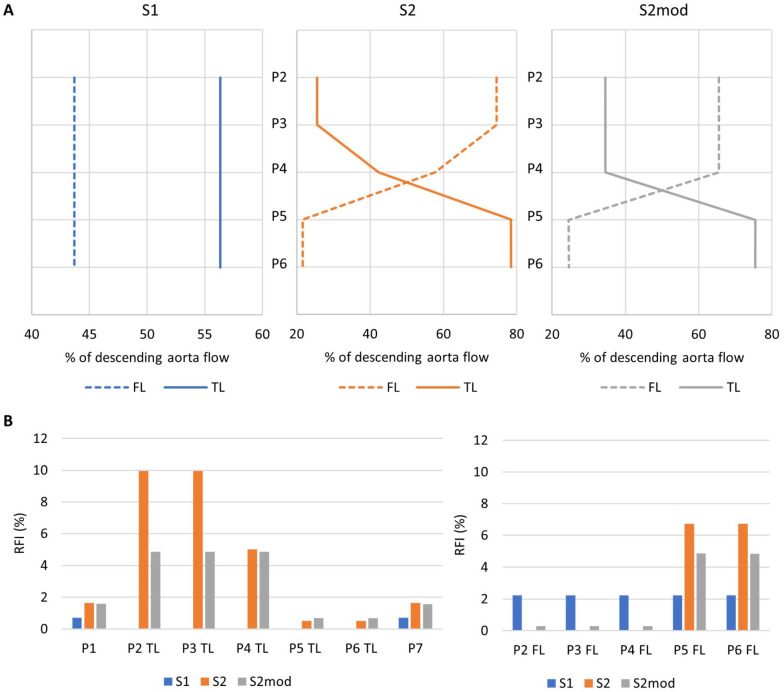
(A) Distribution of descending aorta flow between the TL and FL, measured on planes P2-P6 for models S1, S2, and S2mod. (B) Reverse flow index, calculated on planes P1-P7 for models S1, S2, and S2mod. TL, true lumen; FL, false lumen.

Figure 3A shows the distribution of flow between the TL and FL based on computational simulations. Near the entry tear (plane 2), TL flow was higher than FL flow in S1, but the opposite was observed in S2/S2mod. In S2 and S2mod, moving down the aorta the flow redistributed from the FL to the TL through the re-entry tears. On plane 6, there was higher TL flow in all models. The presence of only 1 additional tear compared with two (S2mod vs S2) resulted in approximately 10% less flow entering the FL and redistributing to the TL; however, the overall trend throughout the aorta did not change.

Figure 3B shows RFI evaluated for all models on all planes. In general, S1 had lower RFI, with a fixed value of 2.2% in the FL, and negligible RFI in the TL. Both S2 and S2mod had higher TL RFI on planes 2 to 4 and higher FL RFI on planes 5 and 6. The number of additional tears greatly affected RFI. The TL RFI was significantly reduced on planes 2 and 3 in S2mod (4.9%) compared with S2 (9.9%), with similar reduction trends seen in FL planes 5 and 6.

The RFI was also calculated within each tear to evaluate change in the direction of flow exchange between the TL and FL. For the entry tear, $Q_{\mathrm{forward}}$ (equation 2) was defined as TL to FL, whereas for re-entry tears $Q_{\mathrm{forward}}$ was defined as FL to TL. For S1, RFI was 2% at both the entry and exit tear. There was no reverse flow at the entry tear for S2 and S2mod, but RFI at the exit tear increased to 5% and 8% for S2mod and S2, respectively. For both S2 and S2mod, there was no reverse flow through the middle re-entry tears, indicating flow was always from FL to TL.

**Figure 4.**
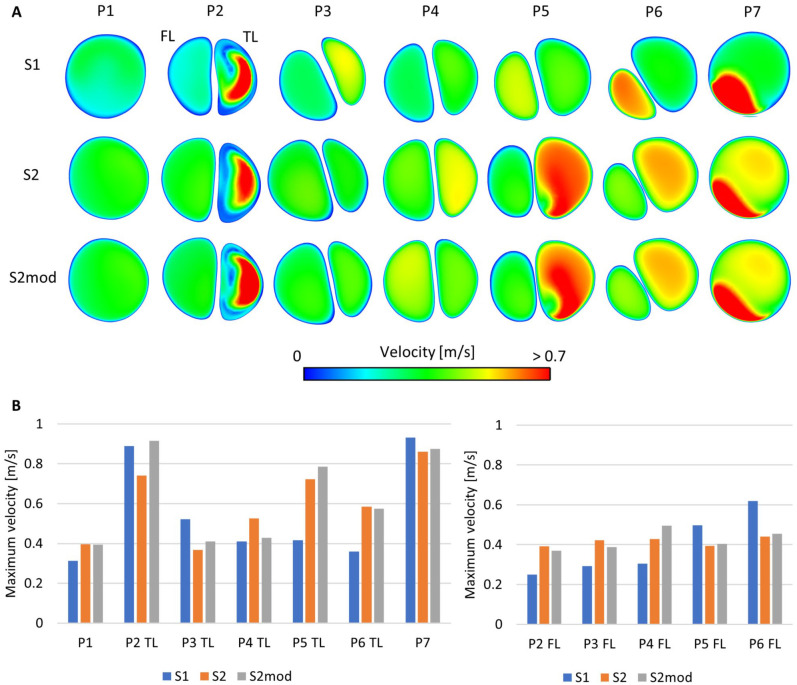
(A) Peak systolic velocity on planes P1-P7 for models S1, S2, and S2mod. (B) TL (left) and FL (right) maximum peak systolic velocity on planes P1-P7, for models S1, S2 and S2mod. TL, true lumen; FL, false lumen.

Figure 4A shows velocity magnitude contours at peak systole for a qualitative comparison of flow patterns, whereas maximum velocities are shown in Figure 4B. Notable differences were observed on plane 4, where S2 and S2mod differed on which lumen had higher maximum velocity, likely due to flow redistribution occurring in S2 but not in S2mod. There were also differences on plane 6 where the effects of flow redistribution were clear, with higher velocities in the TL of S2/S2mod, whereas S1 saw much higher FL velocities. Redistribution effects were also clear on plane 7, where 2 distinctive areas of high velocity were observed in S2/S2mod, whereas in S1 only the high velocity jet from the FL can be seen.

**Figure 5.**
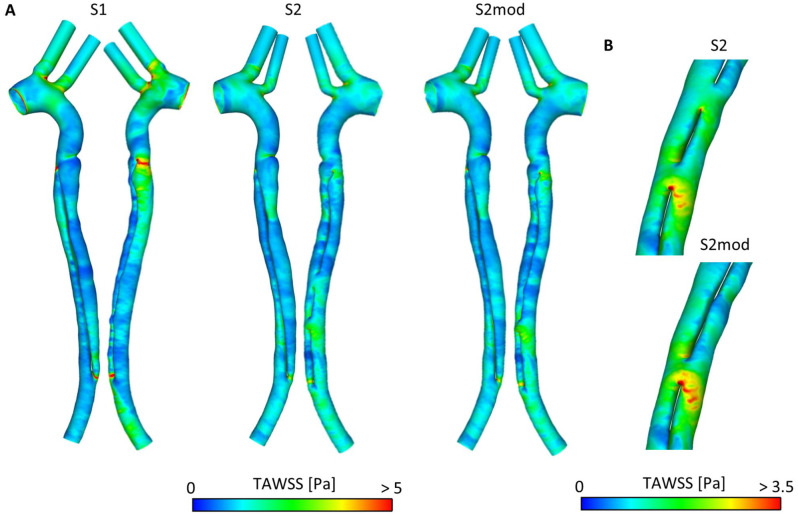
(A) Time averaged wall shear stress (TAWSS) for models S1, S2, and S2mod. (B) TAWSS around re-entry tears in models S2 and S2mod.

The TAWSS maps are shown in Figure 5, where similar patterns can be seen across all models. In the proximal dissection, S1 had the highest TL TAWSS, followed by S2mod and S2 (in line with changes in TL flow volume—Figure 3A). S2mod saw reduced FL TAWSS around the artificially occluded tear and increased TAWSS at the second re-entry tear. Furthermore, S1 saw the highest TAWSS values at the exit tear, compared with S2 and S2mod.

**Figure 6.**
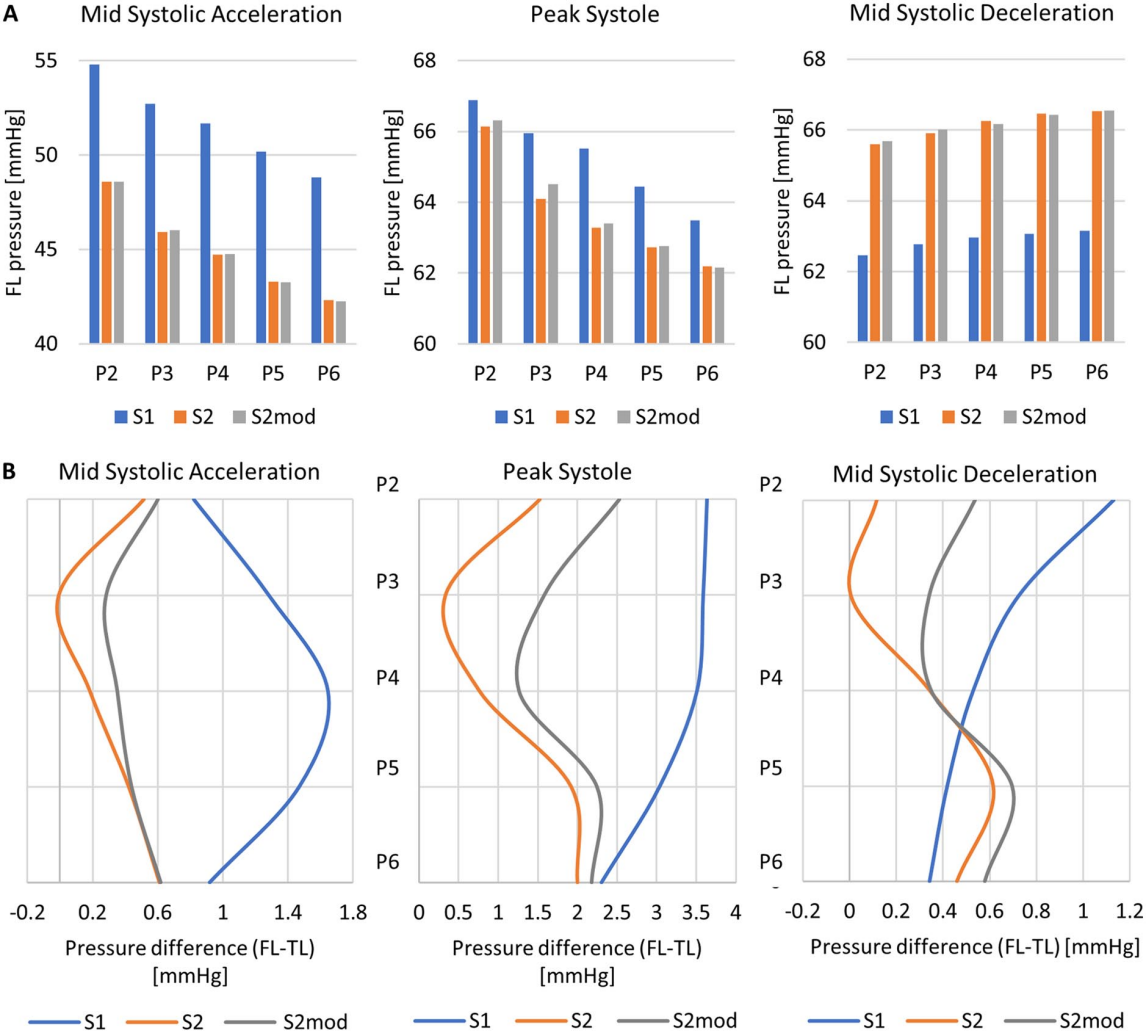
(A) FL pressure and (B) cross-lumen pressure difference, on planes P2-P6 throughout the systolic phase for models S1, S2, and S2mod. TL, true lumen; FL, false lumen.

Pressures in the FL and the FL/TL CLPD are shown in Figure 6. During mid-systolic acceleration and peak systole, S2 and S2mod saw nearly equal pressures, whereas S1 saw higher FL pressures, by up to 6.9 mm Hg. At mid-systolic deceleration, the trend reversed and S1 saw lower pressures, by up to 3.4 mmHg. The average FL pressure over a cardiac cycle was 54.8 mmHg, 50.8 mmHg, and 50.8 mmHg for S1, S2, and S2mod, respectively. In terms of FL/TL CLPD, apart from 2 locations in S2 (where the difference was close to 0) the FL had a higher pressure than the TL in all cases. In the proximal dissection (plane 2), the lowest CLPD was observed in S2. During most of the systolic phase, S1 had the largest CLPD, followed by S2mod and then S2.

## Discussion

The number of re-entry tears has been highlighted as a potential key parameter in dictating the progression of TBAD.^2–4^ Several experimental^7,8,11^ and computational^9,10,12^ studies were conducted to determine how re-entry tears influence aortic hemodynamics and drive the progression of dissection. However, previous work was either confined to idealized geometries or used non-subject-specific boundary conditions, both of which are essential to accurately reproduce blood flow behaviors within the aorta.

Advancing on the aforementioned work, the present study made full uses of realistic geometries, extracted from CT scans, and physiological boundary conditions, derived from 4D-flow MRI data and in vivo pressure measurements. With this combination of data, computational models of TBAD corresponding to specific stages of the disease were built. Our computational results revealed the influence of re-entry tears on aortic hemodynamics, especially on flow redistribution and CLPD.

Our results showed that increasing the number of tears allowed for flow redistribution from the FL to TL. This is consistent with current understanding that re-entry tears provide extra paths for blood flow between the lumen.^9^ Such flow redistribution led to altered velocity patterns in the form of changes in velocity magnitude and RFI. One experimental study^7^ found that re-entry tears significantly reduced RFI in the upper FL and increased TL RFI. This matches our results where the FL RFI decreased from 2.2% to nearly 0% with the introduction of 1 or 2 additional tears, and TL RFI increased as flow redirected from FL to TL. Flow reversal leads to oscillatory shear that in turn can cause elastin degradation,^20^ potentially leading to FL expansion. In a recent study by Burris et al,^21^ FL ejection fraction (FLEF), equivalent to the RFI calculated in our study, was derived from 4D-flow MRI data and their results suggested that increased entry tear FLEF could be a predictor of aortic growth. This is in line with our findings that the entry tear RFI was higher in S1, following which there was FL growth, whereas RFI was zero in S2, after which there was FL reduction.

Pressure within the lumen is an important factor when considering FL expansion. All models saw slightly higher pressure in the FL than TL, with the largest CLPD of ~3 mm Hg in S1. Furthermore, absolute FL pressures were found to be highest in S1 and reduced on average by 4 mm Hg in S2 and S2mod. This is likely the reason why there was slow, uniform aortic growth between the first 3 scans. With an increase in the number of tears, the absolute FL pressure and the FL/TL CLPD reduced. This can also explain the in vivo observations of a reduction in FL growth followed by an eventual reduction in FL volume after the creation of re-entry tears. In addition to FL growth, there was expansion of the distal re-entry tear between S0 and S2. As well as a higher pressure FL, high TAWSS in S1 (Figure 5) may have contributed to this, by wearing down the already damaged vessel wall at the tear point.

Our finding that additional re-entry tears could reduce FL pressure and CLPD is in line with previous experimental and computational studies^8,9,11,12^ and may explain the findings of anatomical studies^3,4^ which suggested that increasing the number of tears might reduce the risk of aortic growth.

Currently, reinterventions to carry out fenestration procedures in TBAD are generally reserved to treating ischemia complications.^22–24^ However, our results show that when FL pressure is higher than TL the creation of additional tears may be beneficial to avoid aortic rupture, reduce FL expansion, and stabilize the condition. Even after TEVAR, there can be cases where patients experience FL expansion, for example, in the unstented abdominal aorta, or due to stent-graft-induced new entry tears.^25^ In these cases, it is not always possible to cover all re-entry tears to reduce FL flow, due to the potential ischemic complications that can occur when major or minor aortic branches are covered. In such a scenario, fenestration may be beneficial to stabilize local expansion.

Although this study demonstrated the benefit of re-entry tears in reducing FL pressure and CLPD, not all patients will present with a patent higher pressure FL—some patients may experience higher TL pressures. FL thrombosis will also alter the hemodynamic environment in the dissected aorta. In cases with FL thrombosis, it is likely that the conditions required to cause FL expansion may not arise and fenestration intervention would be detrimental. The absence of FL thrombus in our swine model meant that its influence on aortic flow, the level of FL perfusion, and pressure distributions could not be assessed. Furthermore, the re-entry tears created in this model were of sufficient size to allow substantial blood exchange between the TL and FL. Studies^10,26^ have shown that tear size influences blood velocities and the direction of flow communication through the tears, and thus different sizes of fenestration may result in different outcomes. Given the single animal model cannot account for all possible disease scenarios, it is emphasized that the conclusions of this study on the potential benefit of fenestration are drawn strictly on a case of a fully perfused patent FL with a higher pressurized FL, where the created fenestrations are large enough to allow for sufficient TL/FL blood exchange.

Therefore, knowledge of TL and FL pressures appears to be crucial in evaluating whether fenestration would be beneficial or not. However, in vivo measurement of intraluminal pressures requires an invasive procedure that cannot be done during routine follow-ups. Although CFD simulations can predict pressure gradients, pressure values are sensitive to boundary conditions, which must be tuned using subject-specific flow measurements.^18,27^ Calculating pressure directly from 4D-flow MRI data^28^ appears to offer a noninvasive option if this method can be applied to TBAD.

The aortic wall is assumed to be rigid in the computational model that may affect the predicted pressure values, with previous studies showing rigid wall simulations can overestimate pulse pressure^29^ and CLPD.^30^ Considering that the TBAD models correspond to conditions at least 9 months after the initiation of dissection when flap mobility is likely to be limited^31^ and the presence of calcification that can also increase flap stiffness, the effect of a rigid wall assumption on simulation results may not be as significant as reported previously. Nevertheless, the implementation of a fluid-structure interaction model would improve the accuracy of predicted pressure.

## Conclusion

Type B aortic dissection can vary significantly across patients. With potentially life-threatening complications arising from the progression of the disease, it is essential to gain a mechanistic understanding of the potential factors that dictate aortic remodeling. Through a longitudinal study of a controlled swine model with combined 4D-flow MRI and computational modeling, we were able to assess the influence of re-entry tears on aortic hemodynamics and understand their role in aortic growth. Our results show that introducing additional re-entry tears, in cases with a higher pressurized fully perfused FL, can be beneficial in reducing CLPD, thus slowing and potentially reversing FL expansion. This has implication for the potential role of fenestration, currently used to treat ischemic complications, in the control of FL expansion in TBAD under certain scenarios and we recommend further exploration using a larger data set.
